# Inspiring impression motivation and fostering knowledge sharing behavior: Role of inclusive leadership for ostracized employees

**DOI:** 10.3389/fpsyg.2026.1793447

**Published:** 2026-03-06

**Authors:** Yang Yang, Ying Li, Yaqin Wang, Hongyuan Lu, Yiyuan Liang

**Affiliations:** 1 School of Mathematics, Southwestern University of Finance and Economics, Chengdu, China; 2 School of International Business, Southwestern University of Finance and Economics, Chengdu, China; 3 School of Guangzhou City Construction College, Guangzhou, China; 4 Institute of Western China Economic Research, Southwestern University of Finance and Economics, Chengdu, China; 5 School of Business, Sichuan University, Chengdu, China

**Keywords:** impression motivation, inclusive leadership, knowledge sharing behavior, two-component model, workplace ostracism

## Abstract

**Introduction:**

Workplace ostracism is generally assumed to diminish employees’ willingness to share knowledge, yet empirical findings remain inconclusive. Drawing on the two-component model of impression management, this research examines whether inclusive leadership influences ostracized employees’ knowledge sharing behavior through impression motivation.

**Methods:**

We conducted two scenario-based experiments (Study 1: hotel employees, *N* = 211; Study 2: software employees, *N* = 247) and a two-wave field survey (Study 3: full-time employees from various professions in China, *N* = 221).

**Results:**

Results across the three studies consistently demonstrated that inclusive leadership moderates the relationship between workplace ostracism and knowledge sharing behavior. When inclusive leadership was low, workplace ostracism negatively affected impression motivation, which in turn reduced knowledge sharing. Conversely, when inclusive leadership was high, workplace ostracism positively influenced impression motivation, subsequently enhancing knowledge sharing behavior. Impression motivation mediated the interactive effect of workplace ostracism and inclusive leadership on knowledge sharing.

**Discussion:**

These findings advance the workplace ostracism literature by identifying inclusive leadership as a critical boundary condition that can transform social exclusion into constructive behavioral outcomes. They also extend the two-component model to the domain of knowledge management and offer practical implications for leadership development in organizations.

## 1 Introduction

In today’s knowledge-intensive organizations, knowledge sharing is widely regarded as a critical behavior for fostering organizational learning, innovation, and sustainable competitiveness (Peng, 2022; Li et al., 2023). Importantly, knowledge sharing is inherently discretionary and socially embedded, relying heavily on employees’ perceptions of interpersonal relationships, social acceptance, and anticipated relational returns (Peng, 2022; Li et al., 2023). In this context, workplace ostracism, defined as the experience of being ignored or excluded by others at work (Ferris et al., 2008), is a salient negative social cue that may undermine employees’ willingness to share knowledge (Gao and He, 2019; Du et al., 2025). Prior research, largely grounded in conservation of resources theory and social exchange theory, generally suggests that workplace ostracism depletes personal resources or disrupts reciprocal relationships, thereby constraining knowledge sharing behavior (Peng, 2022; Du et al., 2025). However, emerging evidence indicates that the behavioral consequences of workplace ostracism are not uniformly negative. Under certain conditions, ostracized employees may engage in effortful and proactive extra-role behaviors rather than disengaging entirely (Chen et al., 2024). This contradiction suggests that the relationship between workplace ostracism and knowledge sharing is likely conditional on key contextual factors that remain underexplored.

Addressing this issue requires identifying contextual cues that shape how ostracized employees interpret exclusionary experiences and evaluate the feasibility of proactive responses. Among various contextual factors, leadership plays a particularly influential role by signaling inclusion, status recognition, and future opportunities within the organization. In this regard, inclusive leadership is especially relevant. By emphasizing openness, accessibility, and appreciation of employees’ contributions, inclusive leadership directly addresses individuals’ needs for belongingness and uniqueness (Korkmaz et al., 2022). Empirical evidence further demonstrates that inclusive leadership is a strong predictor of employees’ perceived insider status, exhibiting greater explanatory power than many other leadership styles (Lin et al., 2025). Moreover, recent research shows that inclusive leadership functions as an important contextual resource in adverse work environments (Siddiquei et al., 2026), suggesting that it may shape whether ostracized employees perceive knowledge sharing as a feasible and worthwhile response to social exclusion.

To theorize how leadership context shapes ostracized employees’ behavioral choices, this study draws on the two-component model of impression management. This model posits that individuals’ responses to social threats depend on impression motivation and impression construction. Impression motivation focuses on whether they wish to manage others’ perceptions, while impression construction determines what behaviors they choose (Leary and Kowalski, 1990). From this perspective, workplace ostracism threatens employees’ social image, but whether this threat translates into proactive behavior depends on contextual cues. Inclusive leadership provides such a cue by signaling the feasibility of impression repair. When inclusive leadership is low, ostracized employees are likely to perceive impression management efforts as ineffective, suppressing impression motivation and knowledge sharing. In contrast, high inclusive leadership signals social recognition, activating ostracized employees’ impression motivation. Knowledge sharing, as a visible and socially valued extra-role behavior, serves as an impression construction strategy through which employees can signal competence and commitment. Accordingly, inclusive leadership moderates the effect of workplace ostracism on knowledge sharing via impression motivation.

In doing so, this research makes three key contributions. First, it advances the workplace ostracism literature by conceptualizing ostracism as a conditional rather than uniformly detrimental social experience, extending prior resource-based and exchange-based explanations. Second, it identifies inclusive leadership as a critical boundary condition that shapes ostracized employees’ behavioral responses, extending research on inclusive leadership and perceived insider status to exclusionary contexts (Korkmaz et al., 2022; Lin et al., 2025). Third, by extending the two-component model of impression management to the context of workplace ostracism and knowledge sharing, this study elucidates the psychological mechanism through which leadership context transforms social exclusion into proactive extra-role behavior.

## 2 Theoretical framework and hypotheses

### 2.1 Two-component model of impression management

Individuals have an ongoing interest in impression management to attain expected rewards in the workplace (Bolino, 1999). In line with this, the two-component model proposes two discrete processes to outline people’s impression management: the first is an impression motivation process, wherein the extent to which people are motivated to control how others see them and the circumstances under which people are motivated to manage their images are considered; the second is an impression construction process wherein people choose to adopt a particular manner over others (Leary and Kowalski, 1990).

In the impression motivation process, the two-component model emphasizes that individuals are more likely to engage in impression management when the impressions they create are highly relevant to valued goals and when influential targets control desired outcomes (Leary and Kowalski, 1990; Richman and Leary, 2009). Workplace ostracism threatens employees’ social identity and sense of belonging, thereby heightening impression-related concerns. However, whether such concerns translate into heightened impression motivation depends on situational signals regarding the feasibility of image repair. Inclusive leadership provides such signals. By emphasizing openness, accessibility, and recognition, inclusive leaders represent a significant target whose approval is perceived as instrumental for social re-inclusion and identity restoration (Carmeli et al., 2010; Nishii and Mayer, 2009). Consequently, under high inclusive leadership, workplace ostracism is more likely to activate impression motivation rather than disengagement.

In the impression construction process, the two-component model identifies three interpersonal factors for behavioral strategies, namely current and potential social image, role constraints, and target values (Leary and Kowalski, 1990). With this approach, knowledge sharing behavior can thus be a good impression management tactic for ostracized employees. Knowledge sharing is a strong proof of competence that can repair employees’ “devalued” image (Bock et al., 2005; Du et al., 2025). Additionally, knowledge sharing can help build strong work relationships with colleagues and help them at work, without threatening their status (Grant and Mayer, 2009; Peng, 2022). Moreover, knowledge sharing behavior pushes organizations to deal with complex environment changes and promotes innovation to not only fit the role constraints of employees, but also meet the expectations of leaders and organizations (Leary and Kowalski, 1990; Hollander, 2009; Carmeli et al., 2010). Thus, for ostracized employees, engaging in knowledge sharing behavior helps them build a competent and friendly image.

Overall, to interpret the effect of the relationship between workplace ostracism and inclusive leadership on the behavior of ostracized employees, we use the two-component model as the theoretical foundation of our impression management model. Through the impression motivation process, inclusive leadership facilitates ostracized employees’ impression motivation. Through the impression construction process, the expectations of the leader and organization, and the role constraints of employees prompt ostracized employees to adopt knowledge sharing behavior.

### 2.2 Hypothesis development

Impression motivation refers to the extent to which individuals are driven to manage how they are perceived by others, particularly when impressions are relevant to valued goals and influential targets control desired outcomes (Leary and Kowalski, 1990). Drawing on the two-component model of impression management, impression motivation is heightened when the impression made is relevant to achieving valued goals, and when a significant target who can facilitate those goals is present (Richman and Leary, 2009). From this perspective, workplace ostracism constitutes a salient social threat that signals devaluation and exclusion, thereby heightening concerns about one’s social image and organizational standing (Peng, 2022). However, the two-component model emphasizes that social threats do not uniformly activate impression motivation; rather, motivation depends on whether individuals perceive impression management efforts as feasible and potentially rewarding given the surrounding context.

Inclusive leadership constitutes a critical contextual cue in this process. Inclusive leaders emphasize openness, accessibility, and recognition, signaling that followers’ efforts are noticed and valued (Korkmaz et al., 2022). Empirical evidence further indicates that inclusive leadership strongly enhances employees’ perceived insider status, underscoring its unique role in facilitating social inclusion and identity restoration (Lin et al., 2025). For ostracized employees, such leaders represent a significant target whose evaluations are highly relevant to regaining acceptance and restoring organizational standing.

When inclusive leadership is not employed, ostracized employees are likely to perceive limited opportunities for successful image repair. In the absence of supportive leadership signals, impression management efforts may be viewed as ineffective or risky, leading employees experiencing high workplace ostracism to disengage rather than invest effort in managing others’ perceptions. Under these conditions, higher levels of workplace ostracism are expected to be associated with lower impression motivation. In contrast, when inclusive leadership is employed, leaders’ openness and recognition signal that social re-inclusion remains attainable. In this context, workplace ostracism heightens the relevance of managing impressions, as employees perceive a viable pathway to restore their image through the inclusive leader. Consequently, employees experiencing workplace ostracism are more likely to exhibit elevated impression motivation compared to those experiencing low workplace ostracism. Accordingly, we propose the following hypothesis:

*H1*: Workplace ostracism and inclusive leadership have an interactive effect on impression motivation. When inclusive leadership is not employed, employees under high workplace ostracism exhibit less impression motivation compared to those under low workplace ostracism; when inclusive leadership is employed, employees under high workplace ostracism demonstrate more impression motivation compared to those under low workplace ostracism.

Knowledge sharing refers to the process in which employees exchange knowledge to cooperate with or help others to jointly solve problems and create additional knowledge (Cummings, 2004). It is a discretionary and socially embedded extra-role behavior that exposes employees to interpersonal visibility and evaluation (Bartol and Srivastava, 2002; Bock et al., 2005). As such, employees’ willingness to share knowledge is highly sensitive to social cues that signal whether such behaviors are likely to be acknowledged and interpreted positively (Peng, 2022; Li et al., 2023). Workplace ostracism, which communicates exclusion and devaluation, therefore has important implications for knowledge sharing, but its effects depend on the broader leadership context.

When inclusive leadership is low, ostracized employees receive few signals that their proactive contributions will be noticed or valued. In this context, engaging in knowledge sharing is unlikely to improve one’s standing or repair a devalued image. Consequently, employees experiencing high workplace ostracism are more likely to refrain from sharing knowledge and limit discretionary contributions compared to those experiencing low ostracism. In contrast, inclusive leadership fundamentally reshapes the social meaning of knowledge sharing. By emphasizing openness, accessibility, and appreciation of employee contributions, inclusive leaders signal that visible, cooperative behaviors are welcomed and valued (Korkmaz et al., 2022). Empirical evidence further indicates that inclusive leadership enhances employees’ perceived insider status, which is closely associated with engagement in extra-role and cooperative behaviors (Lin et al., 2025). Under such conditions, knowledge sharing becomes a viable and socially meaningful way for employees to demonstrate competence and commitment. As a result, for employees experiencing high workplace ostracism, the presence of inclusive leadership may encourage greater engagement in knowledge sharing compared to those experiencing low ostracism. Taken together, we propose that:

*H2*: Workplace ostracism and inclusive leadership have an interactive effect on knowledge sharing behavior. When inclusive leadership is not employed, employees under high workplace ostracism exhibit less knowledge sharing behavior compared to those under low workplace ostracism; when inclusive leadership is employed, employees under high workplace ostracism demonstrate more knowledge sharing behavior compared to those under low workplace ostracism.

According to the two-component model of impression management, once impression motivation is activated, individuals engage in the impression construction process by selecting behaviors that can convey a desired image to others (Leary and Kowalski, 1990). Therefore, when ostracized employees experience a threatened or devalued social image, they must select specific behavioral tactics to construct a desired impression (Richman and Leary, 2009).

Knowledge sharing represents a particularly effective impression construction strategy in this context. First, as a visible and socially valued extra-role behavior, knowledge sharing allows individuals to demonstrate professional competence, contribute to collective goals, and foster positive interpersonal relationships without threatening others’ status (Bock et al., 2005; Hu and Xie, 2015). Second, it functions as a form of affiliative and non-threatening prosocial behavior. Unlike more assertive or challenging forms of citizenship, knowledge sharing fosters collaboration, helps colleagues, and strengthens social bonds without directly threatening others’ status (Grant and Mayer, 2009). This makes it a particularly suitable tactic for rebuilding social acceptance in a manner that is less likely to provoke further resistance or exclusion. Third, knowledge sharing aligns with organizational and leadership values, such as innovation, collaboration, and collective success (Zhang et al., 2011). By engaging in this behavior, ostracized employees can demonstrate alignment with organizational objectives, thereby enhancing their legitimacy in the eyes of both leaders and peers. Accordingly, we propose that:

*H3*: Impression motivation has a positive effect on knowledge sharing behavior.

### 2.3 Integrated model

Based on the two-component model (Leary and Kowalski, 1990), the present study explains how the interactive effect of workplace ostracism and inclusive leadership is transmitted to knowledge sharing behavior through impression motivation. In the impression motivation process, leadership context determines whether workplace ostracism activates efforts to manage social image or instead leads to withdrawal (Lv et al., 2024). Inclusive leadership, by signaling openness and recognition, increases the motivation of ostracized employees to manage image by adopting positive behaviors rather than withdrawal or other antisocial manners (Richman and Leary, 2009). In the subsequent impression construction process, to project a valued and accepted image, the role constraints of ostracized employees and organizational goals encourage them to engage in knowledge sharing behavior as an impression management tactic (Leary and Kowalski, 1990).

Taken together, the two-component model suggests an integrated process in which the interactive effect of workplace ostracism and inclusive leadership first shapes employees’ impression motivation and subsequently guides their engagement in knowledge sharing behavior. Accordingly, impression motivation functions as the psychological mechanism that links the interaction between workplace ostracism and inclusive leadership to knowledge sharing behavior. Thus, we hypothesize the following:

*H4*: Impression motivation mediates the interaction effect of workplace ostracism and inclusive leadership on knowledge sharing behavior.

**FIGURE 1:**
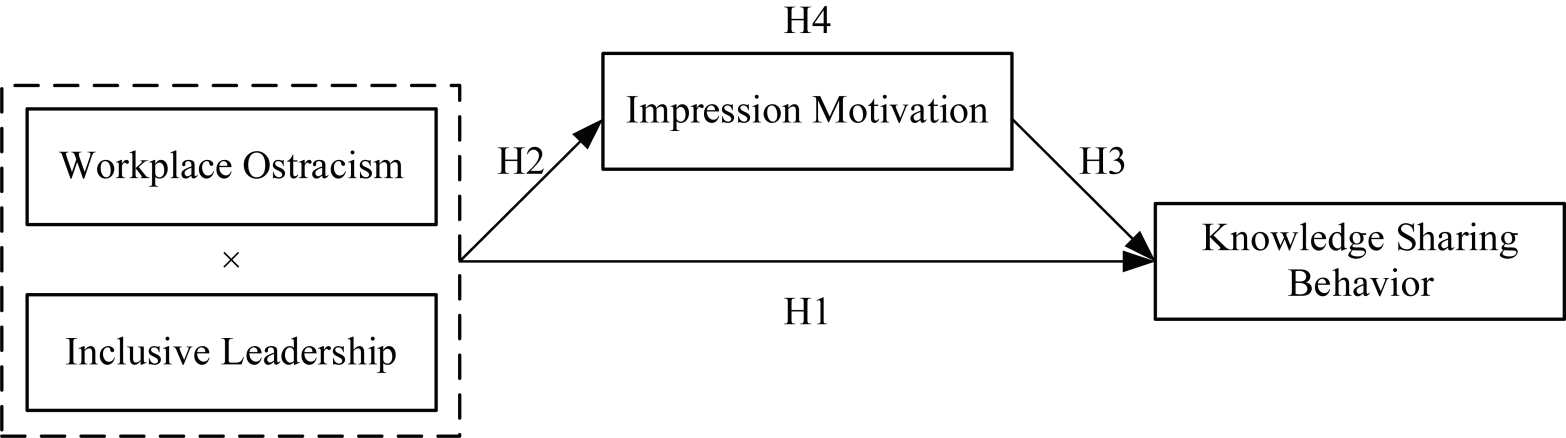
Theoretical model of the current research.

The theoretical model is shown in Figure 1.

## 3 Overview

We conducted three independent studies to test our hypotheses. Study 1 used an experimental design to test the interaction effect of workplace ostracism and inclusive leadership on knowledge sharing behavior. In this study, we manipulated workplace ostracism and the existence of inclusive leadership using a scenario-based study design. The participants of this studies came from the hotel industry. To generalize our results, Study 2 recruited employees from the software industry to test our overall theoretical model and the moderated mediation effect. Study 3 employed a two-wave survey of employees from various professions in China. Using data from different industries to test the research hypotheses can enhance the reliability of the research results. Critically, the research protocols for these three studies were formally reviewed and approved by the Institutional Review Board of the authors’ host institution prior to any data collection. In addition, we complied with APA’s policy of ethical treatment of participants in this study. The experiment and survey process followed the principle of confidentiality informed consent, all participants filled in a unified questionnaire anonymously. This research has received approval from the Experimental Ethics Committee of the Psychology Faculty at Southwestern University of Finance and Economics.

## 4 Study 1

### 4.1 Sample and procedure

Study 1 employed a 2 (Inclusive leadership: not employed vs. employed) × 2 (Workplace ostracism: low vs. high) between-subjects experimental design. In the hospitality industry, employees must collaborate to complete tasks, making the sharing of skills and experiences critically important. Therefore, with the assistance of the human resources department, we contacted a five-star hotel and recruited a total of 211 front-line employees to participate in the experiment (56.4% female, M_age_ = 24.89, SD = 3.753). Participants were randomly assigned to one of the four experimental conditions.

We used the scenario experimental method to direct manipulation of independent variables (Balliet and Ferris, 2013). We conducted workplace ostracism scenario following Ferris et al. (2008), and an inclusive leadership scenario was adopted from Carmeli et al. (2010). After the introduction of the work situation, participants in the workplace ostracism condition read about how other colleagues had excluded the participant. For example, the scenario illustrates, “the team members treated me like I am not there” and “few colleagues invited me to party after work.” In contrast, participants in the non-workplace ostracism condition, read about how other colleagues had included and accepted the participants. Additionally, participants in the inclusive leadership present condition read about how the leader had accepted, tolerated, and respected subordinates. For example, the scenario states, “the leader paid so much attention to subordinates’ needs or questions, and helped them to satisfy their needs or fix their problems.” However, participants in the inclusive leadership absent condition read about how the leader had ignored, and been subjective. Upon reading the experimental materials, participants completed a research questionnaire. A short five-item scale from Bock et al. (2005) was used to measure knowledge sharing behavior (α = 0.93). A sample item is: “I will always provide my manuals and methodologies for members of my organization.” We used a three-item scale to examine the manipulation check of the workplace ostracism manipulation (α = 0.89; Ferris et al., 2008), and adopted a three-item scale to examine the inclusive leader manipulation (α = 0.90; Carmeli et al., 2010). All the items use a 7-point scale (1 = strongly disagree, 7 = strongly agree).

### 4.2 Results

#### 4.2.1 Manipulation check

After controlling for gender, age, and inclusive leadership, the first ANOVA results indicated that participants in the workplace ostracism group experienced significantly higher level of exclusion than those in the non-workplace ostracism group [M_low_ = 2.53, SD = 1.083, M_high_ = 5.19, SD = 0.787; *F*(1, 206) = 407.506, *p* < 0.001, η^2^ = 0.664]. Similarly, after adding gender, age, and workplace ostracism variables into the second model, the second ANOVA results also showed significant variation between inclusive leadership present group and inclusive leadership absent group [M_*not employed*_ = 3.25, SD = 0.718, M_employed_ = 5.27, SD = 0.743; *F*(1, 206) = 395.446, *p* < 0.001, η^2^ = 0.657].

#### 4.2.2 Hypotheses testing

The mean, standard deviation, and correlations of variables are listed in Table 1. Data were analyzed by 2 (Workplace ostracism: low vs. high) × 2 (Inclusive leadership: not employed vs. employed) two-way ANOVA. We controlled gender and age as covariates, and included workplace ostracism, inclusive leadership as predictors of knowledge sharing behavior. The results showed that the main effect of workplace ostracism was significant [*F*(1, 205) = 7.036, *p* = 0.009, η^2^ = 0.033), the main effect of inclusive leadership was significant [*F*(1, 205) = 24.154, *p* < 0.001, η^2^ = 0.105], most importantly, the interaction effect of workplace ostracism and inclusive leadership was significant [*F*(1, 205) = 30.624, *p* < 0.001, η^2^ = 0.130]. Gender [*F*(1, 205) = 0.010, *p* = 0.921, η^2^ < 0.001] and age [*F*(1, 205) = 0.271, *p* = 0.604, η^2^ = 0.001] were none significant relative to knowledge sharing behavior.

**TABLE 1:** Descriptive statistics and correlations (Study 1).

Variable	Mean	SD	1	2	3	4	5
1. Age	24.89	3.753	–	–	(0.93)	(0.89)	(0.91)
2. Gender	0.44	0.497	0.06				
3. Workplace ostracism	3.84	1.632	−0.04	0.01			
4. Inclusive leadership	4.27	1.246	0.04	0.02	0.01		
5. Knowledge sharing behavior	4.62	1.511	0.01	−0.00	−0.20**	0.27**	

*N* = 211. Gender was coded “0” for female, and “1” for male. Values in parentheses on the diagonal are Cronbach’s α coefficients. Values for Workplace ostracism and Inclusive leadership represent manipulation check scores.

**p < 0.01.

**FIGURE 2:**
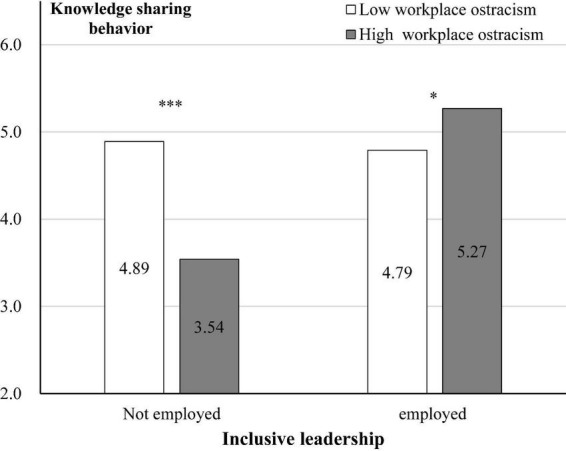
Results of study 1. *p < 0.05. ***p < 0.001.

Further, simple effect tests showed that (Figure 2): In the non-inclusive leadership condition, compared to low workplace ostracism, participants in high workplace ostracism reported a lower level of knowledge sharing behavior [M_low_ = 4.89, SD = 1.251, M_high_ = 3.54, SD = 1.161; *F*(1, 103) = 33.281, *p* < 0.001, η^2^ = 0.244]. In employed inclusive leadership condition, compared to low workplace ostracism, participants in high workplace ostracism reported a higher level of knowledge sharing behavior [M_low_ = 4.79, SD = 1.442, M_high_ = 5.27, SD = 0.899; *F*(1, 104 = 4.198, *p* = 0.043, η^2^ = 0.039].

Using the Bootstrap method (PROCESS, Model 1, *n* = 5,000, confidence interval 95%) to test the moderating effect of inclusive leadership, the results indicated that the interactive effect of workplace ostracism and inclusive leadership was significant [β = 1.854, SE = 0.335, *p* < 0.001, 95% CI = (1.193, 2.514), not included 0]. In the non-inclusive leadership condition, workplace ostracism had a significant negative effect on knowledge sharing behavior (β = −1.370, SE = 0.238, *p* < 0.001, 95% CI = (−1.838, −0.912), not included 0]. In employed inclusive leadership condition, workplace ostracism had a significant positive effect on knowledge sharing behavior [β = 0.484, SE = 0.236, *p* = 0.042, 95% CI = (0.019, 0.948), not included 0]. Thus, Hypothesis 2 was supported.

### 4.3 Discussion

Study 1 used a scenario-based experimental method to test the interactive effect of workplace ostracism and inclusive leadership on employees’ knowledge sharing behavior. The results showed that when inclusive leadership was not employed, compared to low workplace ostracism, high workplace ostracism led employees to engage in less knowledge sharing behavior. This is because in the absence of inclusive leadership, ostracized employees perceive limited opportunities for regaining social inclusion and restoring their image. In the absence of supportive leader cues, impression-management endeavors appear ineffective, which in turn fosters behavioral disengagement over proactive contribution. However, when inclusive leadership was employed, the above relationship reversed. Compared to low workplace ostracism, high workplace ostracism led employees to engage in more knowledge sharing behavior. This reversal can be explained by the signaling function of inclusive leadership, which conveys openness, recognition, and a credible path toward social reaffiliation. In this context, workplace ostracism heightens impression motivation. Consequently, employees turn to knowledge sharing as a strategic means of impression construction. This allows them to demonstrate competence and facilitate reacceptance within the group. Study 1 used a single-industry sample. To enhance the robustness of the conclusions, Study 2 will employ a sample from another industry to re-examine the model. In addition, Study 2 further examined the mediating effect of impression motivation.

## 5 Study 2

### 5.1 Sample and procedure

In Study 2, we collaborated with several local software companies and invited their employees to participate in the experiment. We selected employees from the software industry because their work is highly specialized.. Knowledge sharing is particularly critical for work teams and software companies. A scenario experiment was administered to a sample of full-time employees. Study 2 employed a 2 (Inclusive leadership: not employed vs. employed) × 2 (Workplace ostracism: low vs. high) between-subjects experimental design. Participants were randomly assigned to one of the four conditions.

First, participants read the manipulation materials for workplace ostracism and inclusive leadership. Based on the materials used in Study 1, we adapted the scenarios to fit the software company context. Afterward, participants reported their knowledge sharing behavior (α = 0.94), impression motivation (α = 0.90), workplace ostracism (α = 0.91) and inclusive leadership (α = 0.89). We used the same items for knowledge sharing behavior, workplace ostracism, and inclusive leadership as those in Study 1. We used a four-item scale with Rioux and Penner (2001) to assess impression motivation. A sample item is “I’m working hard, because I want to avoid looking lazy in front of others.” All the items use a 7-point scale (1 = strongly disagree, 7 = strongly agree). All items were translated from English into Chinese using the procedures suggested by Brislin (1980). To demonstrate the robust of our results, as in Study 1, we included gender and age as control variables.

A total of 276 (response rate of 83.6%) employees participated in this experiment. After excluding incomplete responses, 247 valid responses were retained (effective rate of 89.5%). The final sample consisted of 145 males and 102 females, with a mean age of 28.08 years (SD = 3.52).

### 5.2 Results

#### 5.2.1 Confirmatory factor analyses

Table 2 presents means, standard deviations, and bivariate correlations. Before testing the hypotheses, we conducted a series of confirmatory factor analyses (CFA) to evaluate the discriminant validity of our measures. The maximum likelihood estimation procedure was used to estimate model fit. The CFA results showed that the four-factor (i.e., workplace ostracism, inclusive leadership, impression motivation, knowledge sharing behavior) yield an accepted model fit (χ^2^ = 139.265, *df* = 84, χ^2/^ *df* = 1.658, *p* < 0.001; CFI = 0.987, TLI = 0.984, RMSEA = 0.052, SRMR = 0.025). Furthermore, this baseline four-factor model exhibited significantly superior fit compared to all alternative constrained models (e.g., three-factor, two-factor, and single-factor models). These findings provide empirical support for the distinctiveness and discriminant validity of the four core constructs in this study.

**TABLE 2:** Descriptive statistics and correlations (Study 2).

Variable	Mean	SD	1	2	3	4	5	6
1. Gender	0.59	0.493	–	–	(0.89)	(0.91)	(0.90)	(0.94)
2. Age	28.08	3.521	0.06					
3. Inclusive leadership	4.20	1.588	0.03	−0.02				
4. Workplace ostracism	3.89	1.735	−0.01	0.15*	0.03			
5. Impression motivation	4.75	1.223	−0.07	−0.17**	0.29**	−0.04		
6. Knowledge sharing behavior	4.24	1.533	−0.07	−0.17**	0.29**	−0.13*	0.88**	

*N* = 247. Gender was coded “0” for female, and “1” for male. Values in parentheses on the diagonal are Cronbach’s α coefficients. Values for Workplace ostracism and Inclusive leadership represent manipulation check scores.

*p < 0.05.

**p < 0.01.

#### 5.2.2 Manipulation check

After controlling for gender, age, and inclusive leadership, the first ANOVA results indicated that participants in the workplace ostracism group experienced significantly higher level of exclusion than those in the non-workplace ostracism group (M_low_ = 2.53, SD = 1.242, M_high_ = 5.24, SD = .903; *F*(1, 242) = 364.317, *p* < 0.001, η^2^ = .601). Similarly, after adding gender, age, and workplace ostracism variables into the second model, the second ANOVA results also showed significant variation between inclusive leadership present group and inclusive leadership absent group [M_*not employed*_ = 3.05, SD = 1.209, M_employed_ = 5.35, SD = 0.966; *F*(1, 206) = 275.199, *p* < 0.001, η^2^ = 0.532].

#### 5.2.3 Hypotheses testing

Data were analyzed by 2 (Workplace ostracism: low vs. high) × 2 (Inclusive leadership: not employed vs. employed) two-way ANOVA. We controlled gender and age as covariates, and included workplace ostracism, inclusive leadership as predictors of knowledge sharing behavior. The results showed that the main effect of workplace ostracism was not significant [*F*(1, 241) = 2.009, *p* = 0.158, η^2^ = 0.008], the main effect of inclusive leadership was significant [*F*(1, 241) = 24.499, *p* < 0.001, η^2^ = 0.092], most importantly, the interaction effect of workplace ostracism and inclusive leadership was significant [*F*(1, 241) = 17.682, *p* < 0.001, η^2^ = 0.068]. Gender [*F*(1, 241) = 1.496, *p* = 0.223, η^2^ = 0.006] and age [*F*(1, 241) = 2.055, *p* = 0.153, η^2^ = 0.008] were none significant relative to knowledge sharing behavior.

**FIGURE 3:**
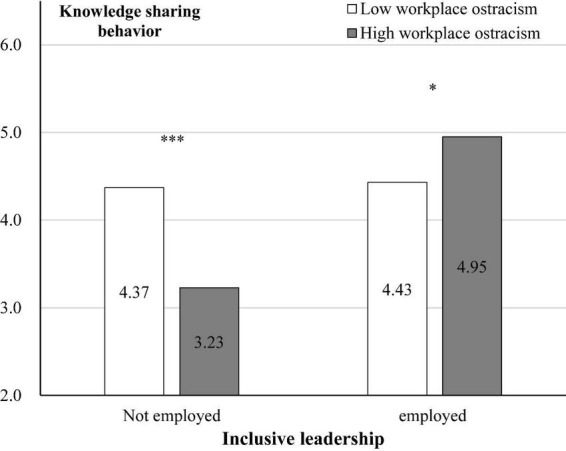
Interaction effect of knowledge sharing behavior. *p < 0.05. ***p < 0.001.

Further, simple effect tests showed that (Figure 3): In the non-inclusive leadership condition, compared to low workplace ostracism, participants in high workplace ostracism reported a lower level of knowledge sharing behavior [M_low_ = 4.37, SD = 1.508, M_high_ = 3.23, SD = 1.284; *F*(1, 121) = 20.231, *p* < 0.001, η^2^ = 0.143]. In employed inclusive leadership condition, compared to low workplace ostracism, participants in high workplace ostracism reported a higher level of knowledge sharing behavior [M_low_ = 4.43, SD = 1.311, M_high_ = 4.95, SD = 1.512; *F*(1, 122) = 4.469, *p* = 0.042, η^2^ = 0.033].

Using the Bootstrap method (PROCESS, Model 1, *n* = 5,000, confidence interval 95%) to test the moderating effect of inclusive leadership, the results indicated that the interactive effect of workplace ostracism and inclusive leadership was significant [β = 1.546, SE = 0.368, *p* < 0.001, 95% CI = (0.822, 2.270), not included 0]. In the non-inclusive leadership condition, workplace ostracism had a significant negative effect on knowledge sharing behavior [β = −1.030, SE = 0.265, *p* < 0.001, 95% CI = (−1.551, −0.509), not included 0]. In employed inclusive leadership condition, workplace ostracism had a significant positive effect on knowledge sharing behavior [β = 0.516, SE = 0.252, *p* = 0.042, 95% CI = (0.019, 1.012), not included 0]. Thus, Hypothesis 2 was supported.

Similarly, we controlled gender and age as covariates, and included workplace ostracism, inclusive leadership as predictors of impression motivation. The results showed that the effect of workplace ostracism was not significant relative to impression motivation [*F*(1, 241) = 0.349, *p* = 0.555, η^2^ = 0.001], the effect of inclusive leadership was significant relative to impression motivation [*F*(1, 241) = 24.802, *p* < 0.001, η^2^ = 0.093], most importantly, the interaction effect of workplace ostracism and inclusive leadership was significant relative to impression motivation [*F*(1, 241) = 10.877, *p* = 0.001, η^2^ = 0.043]. Gender [*F*(1, 241) = 1.630, *p* = 0.203, η^2^ = 0.007] and age [*F*(1, 241) = 3.073, *p* = 0.081, η^2^ = 0.013] were none significant relative to impression motivation.

**FIGURE 4:**
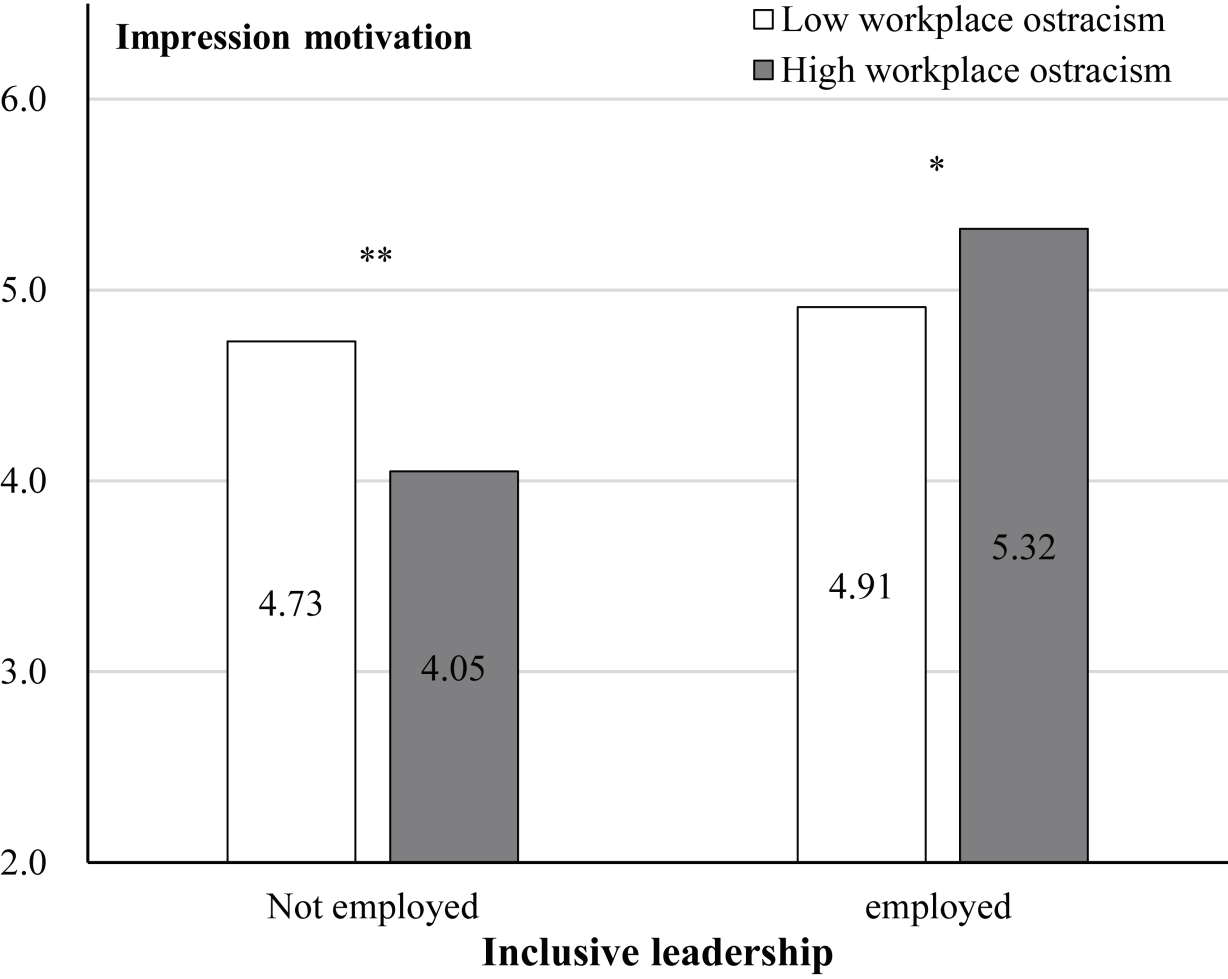
Interaction effect of impression motivation. *p < 0.05. **p < 0.01.

Further, simple effect tests showed that (Figure 4): In the non-inclusive leadership condition, compared to low workplace ostracism, participants in high workplace ostracism reported a lower level of impression motivation [M_low_ = 4.73, SD = 1.187, M_high_ = 4.05, SD = 1.190; *F*(1, 121) = 10.103, *p* = 0.002, η^2^ = 0.077]. In employed inclusive leadership condition, compared to low workplace ostracism, participants in high workplace ostracism reported a higher level of impression motivation [M_low_ = 4.91, SD = 1.073, M_high_ = 5.32, SD = 1.109; *F*(1, 122) = 4.403, *p* = 0.038, η^2^ = 0.035].

Furthermore, we employed the PROCESS macro (Model 7, *n* = 5,000, confidence interval 95%) to test the full model. The results indicated that the interactive effect of workplace ostracism and inclusive leadership was significant positive effect on impression motivation [β = 0.980, SE = 0.297, *p* = 0.001, 95% CI = (0.395, 1.566), not included 0]. Thus, Hypothesis 1 was supported. Moreover, impression motivation had a significant positive effect on knowledge sharing behavior [β = 1.097, SE = 0.039, *p* < 0.001, 95% CI = (1.020, 1.173], not included 0). Thus, Hypothesis 3 was supported.

The results indicated that the moderated mediation effect of workplace ostracism and inclusive leadership on knowledge sharing behavior through impression motivation was significant [Index of moderated mediation = 1.075, SE = 0.351, 95% CI = (0.373, 1.742), not included 0]. In not employed inclusive leadership condition, the indirect effect of workplace ostracism on employees’ knowledge sharing behavior via impression motivation was significantly negative [β = −0.632, SE = 0.254, 95% CI = (−1.127, −0.122), not included 0]. In employed inclusive leadership condition, this indirect effect became significantly positive [β = 0.443, SE = 0.221, 95% CI = (0.006, 0.863), not included 0]. The difference between these conditional indirect effects was 1.075, with a 95% Bootstrap confidence interval of (0.373, 1.742), which did not include 0. Thus, Hypothesis 4 was supported. The direct effect of workplace ostracism on knowledge sharing behavior was not significant [β = −0.151, SE = 0.094, 95% CI = (−0.337, .035), included 0].

### 5.3 Discussion

Study 2 tested the full model and all research hypotheses in another industry. The experimental results showed that when inclusive leadership was not employed, compared to low workplace ostracism, high workplace ostracism led to lower impression motivation and less knowledge sharing behavior. Conversely, when inclusive leadership was employed, compared to low workplace ostracism, high workplace ostracism led to higher impression motivation and more knowledge sharing behavior. Impression motivation mediates the interaction effect of workplace ostracism and inclusive leadership on knowledge sharing behavior. The participants of Study 1 and Study 2 came from the hotel industry and the software industry, respectively. The findings from both studies supported all research hypotheses, demonstrating the robustness of the results. Although scenario-based experiments allow for good control over potential confounding factors, they may lack ecological validity because the scenarios do not reflect participants’ real work experiences. To address this limitation, Study 3 tested the research hypotheses again by surveying employees’ actual work experiences.

## 6 Study 3

### 6.1 Sample and procedure

Study 3 utilized a two-wave survey design involving full-time employees from a wide range of professional backgrounds in China, such as education, engineering, public service, and marketing. To ensure a diverse and representative sample, participants were recruited through a combination of online and offline channels. In the first wave (Time 1), survey was distributed to 410 employees, a total of 320 (response rate of 78%) employees returned the completed survey. More than a week later, these employees were invited to participate the second-wave survey (Time 2). After excluding incomplete responses and invalid questionnaires that did not pass the attention check, a total of 221 employees (response rate of 69.1%) returned completed surveys. The final sample consisted of 88 males and 133 females, and 93.2% were over 25 years, and 78.7% had the organizational tenure over 5 years. They all have associate degree or above.

Measures were adopted in the same manner as in Study2. To demonstrate the robust of our results, we included gender, age, education, and tenure, as control variables. We employed a two-wave time-lagged design with an interval of more than 1 week for several strategic reasons. First, collecting all variables at Time 1—including the workplace ostracism, inclusive leadership, impression motivation, and knowledge sharing behavior—allows us to establish a baseline for each construct, which is essential for conducting a rigorous lagged-effects analysis. Second, by measuring the impression motivation and knowledge sharing behavior again at Time 2, we can examine how the interaction between workplace ostracism and inclusive leadership at Time 1 predicts subsequent changes in employees’ motivations and behaviors. This temporal separation helps to mitigate common method variance and provides a clearer window into the psychological processes that unfold over time (Podsakoff et al., 2003). Specifically, it allows us to demonstrate that the hypothesized interaction effect at Time 1 is not merely a cross-sectional correlation but a precursor to the shifts in impression motivation and knowledge sharing observed after more than 1 week.

### 6.2 Results

Table 3 presents means, standard deviations, and bivariate correlations. Before testing the hypotheses, various procedures have been conducted to address common method bias. Firstly, Harmon’s one-factor test was employed. An unrotated factor solution was subjected to factor analysis, revealing that the initial factor accounted for 38.363% of the total variance, falling short of the established threshold of 40%. Secondly, Mplus 8.0 software was utilized to examine the proposed relationships. The CFA results showed that the six-factor (i.e., workplace ostracism at Time 1, inclusive leadership at Time 1, impression motivation at Time 1 and Time 2, knowledge sharing behavior at Time 1 and Time 2) yield an accepted model fit (χ^2^ = 609.847, *df* = 303, χ^2/^ *df* = 2.013, *p* < 0.001; CFI = 0.901, RMSEA = 0.068, SRMR = 0.057). Furthermore, this baseline four-factor model exhibited significantly superior fit compared to all alternative constrained models (e.g., three-factor, two-factor, and single-factor models). These findings provide empirical support for the distinctiveness and discriminant validity of the four core constructs in this study.

**TABLE 3:** Descriptive statistics and correlations (Study 3).

Variable	*Mean*	*SD*	1	2	3	4	5	6	7	8	9	10
1. Gender	0.60	0.491	–	–	–	–	(0.81)	(0.80)	(0.84)	(0.86)	(0.84)	(0.90)
2. Age	2.52	0.685	0.01									
3. Education	1.86	0.474	0.06	−0.12								
4. Tenure	3.05	0.712	−0.07	0.71**	−0.03							
5. Workplace ostracism (T1)	1.97	0.894	−0.05	−0.16*	0.17**	−0.07						
6. Inclusive leadership (T1)	3.88	0.827	−0.08	0.03	0.10	−0.05	0.04					
7. Impression motivation (T1)	2.90	1.085	0.03	−0.05	0.08	−0.07	−0.06	−0.15*				
8. Impression motivation (T2)	3.25	1.104	0.00	0.01	−0.02	0.03	−0.16*	−0.35**	0.58**			
9. Knowledge sharing behavior (T1)	3.86	0.719	0.08	0.03	0.05	−0.02	−0.31**	0.19**	0.34**	0.28**		
10. Knowledge sharing behavior (T2)	3.98	0.831	0.02	0.13*	−0.18**	0.09	−0.47**	−0.01	0.15*	0.40**	0.52**	

*N* = 221 employees. Gender was coded “0” for male, and “1” for female. Age was coded “1” for 25 years or under, “2” for 26–35 years, “3” for 36–45 years, and “4” for 45 years or above. Education was coded “1” for master degree, “2” for bachelor degree and “3” for college degree. Tenure was coded “1” for 1 year or under, “2” for 1–5 years, “3” for 5–10 years, “4” for over 10 years. T1 = variables rated at Time 1. T2 = variables rated at Time 2. Values in parentheses on the diagonal are Cronbach’s alpha coefficients.

*p < 0.05.

**p < 0.01.

We employed the PROCESS (Model 7, *n* = 5,000, confidence interval 95%) to test the full model. We controlled gender, age, education, tenure, and Time 1 impression motivation. The interaction of Time 1 workplace ostracism and Time 1 inclusive leadership had a significant positive effect on Time 2 impression motivation [β = 0.435, SE = .073, *p* < 0.001, 95% CI = (0.291, 0.579), not included 0]. Thus, Hypothesis 1 was supported. Similarly, We controlled gender, age, education, tenure, and Time 1 knowledge sharing behavior. The interaction of Time 1 workplace ostracism and Time 1 inclusive leadership had a significant positive effect on Time 2 knowledge sharing behavior (β = 0.311, SE = 0.058, *p* < 0.001, 95% CI = (0.195, .427), not included 0]. Thus, Hypothesis 2 was supported. Moreover, impression motivation had a significant positive effect on knowledge sharing behavior [β = 0.268, SE = 0.048, *p* < 0.001, 95% CI = (0.172, .363), not included 0]. Thus, Hypothesis 3 was supported.

The results indicated that the moderated mediation effect of workplace ostracism and inclusive leadership on knowledge sharing behavior through impression motivation was significant [Index of moderated mediation = 0.116, SE = 0.042, 95% CI = (0.041, 0.204), not included 0]. In not employed inclusive leadership condition, the indirect effect of workplace ostracism on employees’ knowledge sharing behavior via impression motivation was significantly negative [β = −0.097, SE = 0.036, 95% CI = (−0.172, −0.033), not included 0]. In employed inclusive leadership condition, this indirect effect became significantly positive [β = 0.078, SE = 0.035, 95% CI = (0.018, 0.153), not included 0]. Furthermore, pairwise contrasts of the indirect effects at different levels of the moderator revealed a significant difference between high and low levels of inclusive leadership [Contrast = 0.174, 95% CI = (0.061, 0.306), not included 0]. This further confirms that as the level of inclusive leadership increases, the indirect effect of workplace ostracism on knowledge sharing behavior, mediated by impression motivation, undergoes a significant crossover reversal from negative to positive.

## 7 Conclusion and discussion

### 7.1 Conclusion

Drawing on the two-component model of impression management, this study employed two scenario-based experiments and a two-wave survey to examine whether and how workplace ostracism influences employees’ knowledge sharing behavior under inclusive leadership. Across studies, the findings revealed that workplace ostracism does not exert a uniform effect on knowledge sharing behavior. Instead, its influence depends on the presence of inclusive leadership. When inclusive leadership was low, workplace ostracism was associated with reduced impression motivation and lower levels of knowledge sharing behavior. In contrast, when inclusive leadership was high, workplace ostracism was associated with heightened impression motivation and increased knowledge sharing behavior. Furthermore, impression motivation mediated the interactive effect of workplace ostracism and inclusive leadership on knowledge sharing behavior, indicating that changes in employees’ motivation to manage their social image explain the conditional behavioral outcomes.

These findings help clarify the mixed conclusions reported in prior research on workplace ostracism. Existing studies grounded in conservation of resources theory and social exchange theory have largely emphasized its detrimental consequences for counterproductive behaviors (Peng and Zeng, 2016; Yang and Treadway, 2018), such as knowledge sharing (Gao and He, 2019). The present results are consistent with this view under conditions of low inclusive leadership, where ostracism reduced motivation and behavioral engagement. At the same time, our findings align with emerging evidence suggesting that workplace ostracism may, under certain contextual conditions, elicit constructive responses rather than withdrawal (Romero-Canyas et al., 2010; Cuadrado et al., 2015). By demonstrating that leadership context shapes how employees interpret and respond to exclusionary experiences, this study provides a conditional explanation that reconciles these seemingly inconsistent streams of findings.

### 7.2 Theoretical contributions

Our findings contribute to the literature in several ways. First, building on two-component model, the present study adopts a new perspective to advance a more thoroughly understanding of the effects of workplace ostracism. Prior research mostly explored the negative effect of workplace ostracism from the resource-based perspective (Balliet and Ferris, 2013), which cannot explain the positive effect of workplace ostracism (Cuadrado et al., 2015; Jiang and Zhang, 2021). Rather, as a status threaten, workplace ostracism is highly associated with people’s impression motivation (Richman and Leary, 2009). The present research introduces the impression motivation as the mediator based on the two-component model, and identifies the influence factor of ostracized employees’ impression motivation, explaining under what condition workplace ostracism could produce positive. Hence, from the impression management view, this research complements the existing perspectives, not only reconciling the mixed findings on the effect of workplace ostracism, but also identifying an upside to workplace ostracism.

Second, our study extends prior work by focusing on negative workplace contexts as antecedents of knowledge sharing behavior, and provides a deeper understanding of why negative accident can encourage knowledge sharing behavior Prior research has focused on social exchange theory to consider reciprocal relations as the antecedents of knowledge sharing behavior (Balliet and Ferris, 2013). Yet the principles did not hold in negative workplace context. From the social dilemma perspective (Lu and Tu, 2018), we view engaging in knowledge sharing behavior not as the consequence of relation, but a strategy to construct people’s impression. By considering knowledge sharing behavior as a tactic to reconstruct ostracized employees’ devalued image, this research offers a new perspective on the role of knowledge sharing behavior in negative workplace scenarios, and empirically builds a bridge between negative workplace ostracism and employee extra-role behaviors.

Third, our research responds to the call to consider boundary conditions when studying workplace ostracism (Chen et al., 2017). Although previous research has mainly focused on bilateral ties to explain the effects of workplace ostracism (Yang and Treadway, 2018; Gao and He, 2019), which has demonstrated the link between workplace ostracism and other individual characteristics (Balliet and Ferris, 2013), workplace context moderators have largely been overlooked to date. By viewing workplace ostracism as a multilateral relationship, our impression management model highlights inclusive leadership as a crucial contextual factor capable of triggering the positive effect of workplace ostracism on knowledge sharing behavior, providing a new research avenue for future studies on workplace ostracism as well as suggesting ways to deal with its negative effects.

### 7.3 Practical implications

Our work offers several practical implications. Our findings suggest that inclusive leadership can help promote ostracized employees’ extra-role behaviors. Thus, organizations should strengthen their leadership selection processes, focusing not only on the performance of leaders but also on their ability to cultivate psychological capital and promote psychological safety among employees (Clapp-Smith et al., 2008; Carmeli and Schaubroeck, 2008; Tu et al., 2018). Based on the nature of inclusive leadership, organizations should regard openness, accessibility, and availability as requisite characteristics among leaders.

In addition, organizations should put more emphasis on status stability. For employees who feel accepted in their organization, leaders should maintain a stable and secure organizational environment, ensuring that they do not perceive their status as being threatened (Marr and Thau, 2014). In contrast, for employees who feel excluded in their organization, leaders should create a relatively flexible environment, allowing them to perceive that it is possible to improve the negative impression made on others, and provide opportunities and resources to help them regain approval (Hu and Xie, 2015).

### 7.4 Limitations and future research directions

There may be concerns about the use of college students as participants in our first study; however, studies using students as participants have obtained interesting and valid results. Although the behaviors of students cannot completely represent employee responses, the use of a scenario-based experimental design along with a two-wave survey conducted among full-time employees demonstrated the robustness of our findings (Balliet and Ferris, 2013). Another concern may be that we recruited the same employees in the two waves of the second survey, which may raise potential concerns about common method bias (Podsakoff et al., 2003). However, we used a two-wave design and explored the interaction effect of workplace ostracism and inclusive leadership on knowledge sharing behavior, which mitigated common method bias to some extent (Podsakoff et al., 2003). Nevertheless, a longitudinal study conducted using a different sample of employees could test the model more accurately.

Furthermore, our two studies were conducted within a single culture in China. Compared with Western cultures, “face culture” in China plays a much more important role. This may cause Chinese employees to pay more attention to how others perceive them (Chen et al., 2013; Chen et al., 2017). China is a country with a high-power distance culture (Hofstede, 2001); individuals in this type of culture are more likely to accept their leaders’ decisions and integrate their leaders’ views and attitudes. Conversely, in low power distance cultures, individuals have such a strong sense of self-determination and democracy that their attitudes toward a certain employee will not be affected by how leaders view that employee. Therefore, China’s cultural characteristics may strengthen the effect of inclusive leadership on ostracized employees’ knowledge sharing behavior. Thus, to verify the ecological validity of our studies, future studies should test our model in other cultural contexts characterized by low power distance.

Beyond cultural context, future research may further examine additional individual and situational contingencies that shape the activation of impression motivation in response to workplace ostracism. From the perspective of the two-component model, impression motivation depends on both the perceived relevance of social evaluation and the feasibility of image repair. Individual differences such as proactive personality or emotional intelligence may influence how strongly employees interpret ostracism as a status threat and how effectively they identify significant evaluative targets. Similarly, organizational factors such as team interdependence, performance evaluation systems, or reward structures may alter the perceived utility and visibility of knowledge sharing as an impression construction strategy. By incorporating these individual and contextual variables, future research can extend the current framework and further clarify the boundary conditions under which workplace ostracism leads to constructive.

Finally, based on the impression management model, our study proposed that inclusive leaders could encourage ostracized employees to share their knowledge because ostracized employees want to construct a desired image and “look good” (Bolino, 1999). When ostracized employees regain approval and improve how others perceive them, they may stop sharing their knowledge, which is not what their organization expects. However, there are three motivations that encourage employees to engage in extra-role behaviors, namely impression motivation, prosocial value motivation, and organizational concern motivation (Rioux and Penner, 2001). Here, “looking good” is only an external motivation. Hence, future research should explore how to transform impression motivation into autonomous motivation to allow knowledge sharing behavior to continue relative to external motivation (Gagné et al., 2019).

## Data Availability

The original contributions presented in this study are included in this article/supplementary material, further inquiries can be directed to the corresponding author.
